# Synthesis and Properties of Targeted Radioisotope Carriers Based on Poly(Acrylic Acid) Nanogels

**DOI:** 10.3390/pharmaceutics13081240

**Published:** 2021-08-11

**Authors:** Małgorzata Matusiak, Beata P. Rurarz, Sławomir Kadłubowski, Marian Wolszczak, Urszula Karczmarczyk, Michał Maurin, Beata Kolesińska, Piotr Ulański

**Affiliations:** 1 Institute of Applied Radiation Chemistry, Faculty of Chemistry, Lodz University of Technology, Wróblewskiego 15, 93-590 Lodz, Poland; malgorzata.matusiak.1@p.lodz.pl (M.M.); beata.rurarz@dokt.p.lodz.pl (B.P.R.); slawomir.kadlubowski@p.lodz.pl (S.K.); marian.wolszczak@p.lodz.pl (M.W.); 2 National Centre for Nuclear Research, Radioisotope Centre POLATOM, Andrzeja Sołtana 7, 05-400 Otwock, Poland; urszula.karczmarczyk@polatom.pl (U.K.); michal.maurin@polatom.pl (M.M.); 3 Institute of Organic Chemistry, Faculty of Chemistry, Lodz University of Technology, Żeromskiego 116, 90-924 Lodz, Poland; beata.kolesinska@p.lodz.pl

**Keywords:** radiation chemistry, biomaterials, poly(acrylic acid), biomedical applications, nanocarriers, nanogels, DMTMM, conjugation, bombesin, DOTA, radioisotopes

## Abstract

Radiation crosslinking was employed to obtain nanocarriers based on poly(acrylic acid)—PAA—for targeted delivery of radioactive isotopes. These nanocarriers are internally crosslinked hydrophilic macromolecules—nanogels—bearing carboxylic groups to facilitate functionalization. PAA nanogels were conjugated with an engineered bombesin-derivative—oligopeptide combined with 1,4,7,10-tetraazacyclododecane-1,4,7,10-tetraacetate chelating moiety, aimed to provide selective radioligand transport. 4-(4,6-Dimethoxy-1,3,5-triazin-2-yl)-4-methylmorpholinium (DMTMM) toluene-4-sulfonate was used as the coupling agent. After tests on a model amine—p-toluidine—both commercial and home-synthesized DOTA-bombesin were successfully coupled to the nanogels and the obtained products were characterized. The radiolabeling efficiency of nanocarriers with ^177^Lu, was chromatographically tested. The results provide a proof of concept for the synthesis of radiation-synthesized nanogel-based radioisotope nanocarriers for theranostic applications.

## 1. Introduction

In recent years targeted nanotherapeutics have attracted significant attention. Many carriers as well as targeting ligands have been intensively explored to enable reaching tumor cells in tailored, precise way. A growing body of evidence supports the feasibility of this approach, and an increasing number of formulations is entering clinical trials [1,2,3,4]. Such formulations should be designed in a way that assures that following functionalities are introduced: therapeutic moiety—a delivery vehicle or protein, carrying the therapeutic cargo; targeting ligand—a specific probe, e.g., a small organic molecule, peptide, or antibody able to bind with specific components of the cell, facilitating transport of the construct into the target site [5,6]. The application of nanocarriers allows for the improvement of therapeutic agents’ pharmacokinetics, alters hydrophobic drug solubility in water-based formulations, and alleviates their degradation and clearance by the reticuloendothelial system (RES) components.

One of the most interesting candidates for carriers in targeted nanotherapy are nanogels—spherical polymer networks of internally crosslinked macromolecules with high water content [7]. The open structure of such “frozen coils” is permeable for water molecules and low-molecular-weight solutes. This provides the opportunity for the transport of biologically active compounds in a fluid-like manner, while protecting them against decomposition and ameliorating the side effects of the treatment [8,9,10,11]. Nanogels have drawn significant attention also because of their exceptional biocompatibility, high stability, and relatively easy tuning of size, surface properties, softness, and degradability by altering the chemical composition [12,13]. The incorporation of particular functional groups in the crosslinked polymer structure can induce sensitivity of the particle to environmental factors (e.g., pH, temperature, ionic strength, or light); hence, external stimuli may lead to the controlled release of therapeutic cargo transported by the construct [12,14]. Moreover, groups like -OH, -COOH, -SH, or -NH_2_ enable convenient bioconjugation of targeting ligands [14,15,16,17].

There is a myriad of methods for nanogel preparation, in which physically or chemically crosslinked structures can be obtained [18,19]. Among them, radiation-induced synthesis is particularly attractive. It allows the crosslinking reaction to be carried out without adding harmful initiators or crosslinking agents, rendering the obtained nanoparticles suitable for biomedical applications. This method, exploited by our research group for more than 20 years, allows nanogels to be synthesized from various polymers [20,21,22,23,24,25,26,27]. Poly(acrylic acid) (PAA) is of particular importance. Linear and crosslinked PAA is a component of a number of approved drugs, including ocular drops. Its potential as a material for biomedical application is based on the presence of carboxylic groups: They provide good water solubility, responsiveness of the material to changing pH, and an opportunity for convenient bioconjugation. Moreover, it was shown that the interaction of poly(acrylic acid) with lipid bilayers may destabilize those layers and make them more prone to leakage in a pH-dependent manner. This phenomenon can play an important supporting role in the endosomal escape of the structures based on PAA [28,29,30,31,32]. Nanogels bearing carboxylate groups, synthesized by a similar procedure as the one used in this work, have been demonstrated to be promising nanocarriers for certain drugs [33], oligonucleotides [34], and siRNA [17]. Moreover, it has been shown that such nanogels are capable of bypassing the cell membranes and also crossing the blood–brain barrier while carrying a drug used in therapy for Alzheimer’s disease [35,36].

Conjugation of targeting ligands or therapeutic agents to the carboxylic group-bearing nanocarriers is frequently used for nanotherapeutics synthesis [37,38,39,40,41,42,43,44]. It allows for the formation of both cleavable pH-sensitive connections via ester bonds [45,46], as well as permanent stable amide linkage, which is particularly interesting in the case of biologically active molecules. Condensation of primary amines with nanoparticle carboxylic groups is most commonly performed using carbodiimide chemistry based on 1-ethyl-3-(3-dimethylaminopropyl)carbodiimide (EDC) and *N*-hydroxysuccinimide (NHS)/*N*-hydroxysulfosuccinimide (sulfo-NHS) [47]. Yet, this field can still benefit from further investigation, as there are limited chemical groups that are able to specifically react with carboxylic acids [48]. Recently there has been some attention paid to the other class of coupling agents based on organic triazine derivatives. First, and one of the most popular of this class, is (4,6-dimethoxy-1,3,5-triazin-2-yl)-4-methyl-morpholinium (DMTMM) chloride, which was first reported in 1999 by Kunishiwa et al. [49] and since then it has been primarily used as an amide coupling agent in organic chemistry, particularly in peptide synthesis [50]. Since its first applications, some improvements have been proposed to DMTMM chemistry, mainly by the optimization of the tertiary amine and counterion of the compound [51,52].

Successful application of DMTMM in novel nanotherapeutics development is becoming prominent and an increasing number of nanocarriers is shown to be compatible with *N*-triazinylammonium chemistry. Numerous works indicate the versatility of this approach, allowing for peptides to be coupled to various polymeric carriers, e.g., hyaluronic acid [44], PEG-based block copolymers linked to near-infrared dye IR-780 with photothermal and photodynamic properties [53], and poly-l-glutamic acid [54]. Conejos-Sánchez et al. [55] examined multiple conjugation chemistries for amide linkage formation and found DMTMM activation to be the most reproducible. They used two variants of the compound with various counterions (Cl^−^ and BF_4_^−^) to attach doxycycline to their PGA drug carrier. Moreover, they successfully incorporated the chelating 1,4,7,10-tetraazacyclododecane-1,4,7,10-tetraacetate (DOTA) moiety in their polymer–drug conjugate for radioactive labeling with a positron-emitting ^68^Ga radionuclide.

DOTA is a well-known chelate for lanthanide ions. The resulting chelated complexes have several applications in medicine, e.g., they are used as cancer treatment drugs or contrast agents. When considering the use of DOTA chelators in cancer diagnosis, they are commonly used as a chelating agent connected to monoclonal antibodies (e.g., Yttrium (^90^Y) clivatuzumab tetraxetan [56], Yttrium (^90^Y) tacatuzumab tetraxetan [57]), and small peptides that have an affinity for specific receptors (e.g., DOTATOC, DOTA-TATE [58], DOTA-biotin [59]). There is also a well-known application of DOTA in an MRI contrast agent under the name of gadoteric acid, in which Gd^3+^ is used with the DOTA complex [60].

The incorporation of radioactive components turned out to be a very attractive direction in targeted nanotherapeutics development, and functional nanoparticles labeled with radionuclides have been proposed as a new category of materials. They offer the possibility to exploit the already established principles of nuclear medicine. At the same time, they are also taking advantage of targeting methods as well as other therapeutic and diagnostic modalities elaborated to maximize the effects of treatment with nanomedicine [6]. This novel class of nanoparticulate formulations became a scope of a brand new field of medical science—radionanomedicine [61].

Numerous challenges are in the way of using targeted radionanotherapeutics in the clinic—they have to satisfy the requirements both for nanotherapeutics and radiopharmaceuticals. The final products must ensure high specific activity and sufficient radiostability, achieved by quick, efficient, and safe labeling, and excellent biocompatibility and biodistribution of the nanocarrier is essential. Despite the complex nature of the matter, many researchers are exploring radionanomedicine with promising results [62,63].

The objective of this paper is to present a novel approach to the development of nanoscale carriers based on poly(acrylic acid) nanogels for the targeted delivery of cancer diagnostic and therapeutic agents. The developed carriers are expected to be a common platform to deliver radionuclides for cancer diagnosis and therapy. A selective targeting ability was introduced to the construct by the use of a specially modified targeting ligand—bombesin derivative. The binding region of this peptide shows agonistic affinity to the gastrin-releasing peptide receptor (GRPR), abundantly expressed in many neoplastic conditions, such as breast, prostate, or pancreatic cancer. In this work, the DOTA-bombesin derivative is applied as a chelator for a wide range of radionuclides, e.g., lutetium-177, yttrium-90, and gallium-68, which, with high target specificity, can detect and treat cancer cells. Furthermore, in the future approach, DOTA chelators as an independent derivative can modify the surface of the nanoparticles to achieve a higher radiation dose by more efficient radiolabeling. As an alternative use, DOTA chelators may be labelled with Gd, commonly used in magnetic resonance as a contrast agent, or the surface can be modified by the attachment of fluorophores for optical imaging.

The developed nanocarrier platform allowed radiolabeled nanogels to be obtained, characterized by the radiochemical yield of 99.0 ± 1.1% and specific activity of 0.77 [GB mg^−1^]. Thus, this preliminary study provides a proof-of-concept for the synthesis and functionalization of radiation-derived nanogels for theranostic applications. However, additional studies will be performed to optimize the nanogel radionuclide loading content and their specific activity.

## 2. Materials and Methods

### 2.1. Materials

The following chemicals were used for nanogel synthesis and analysis of the physicochemical properties: poly(acrylic acid) (PAA) of nominal weight-average molecular weight 4.5 × 10^5^ Da (Sigma-Aldrich, St. Louis, MO, USA), sodium hydroxide (NaOH, pure PA, POCH SA), perchloric acid (HClO_4_, 70%, Sigma-Aldrich), and sodium perchlorate (NaClO_4_·H_2_O, Sigma-Aldrich). Solutions were prepared using TKA-Micropure filtered water and ULC/MS-grade water from Biosolve with high purity and high UV transmission. Argon (5.0, Ar ≥ 99.999%, Linde Gaz Polska, Kraków, Poland) was used to saturate the solutions prior to irradiation.

**Scheme 1:**
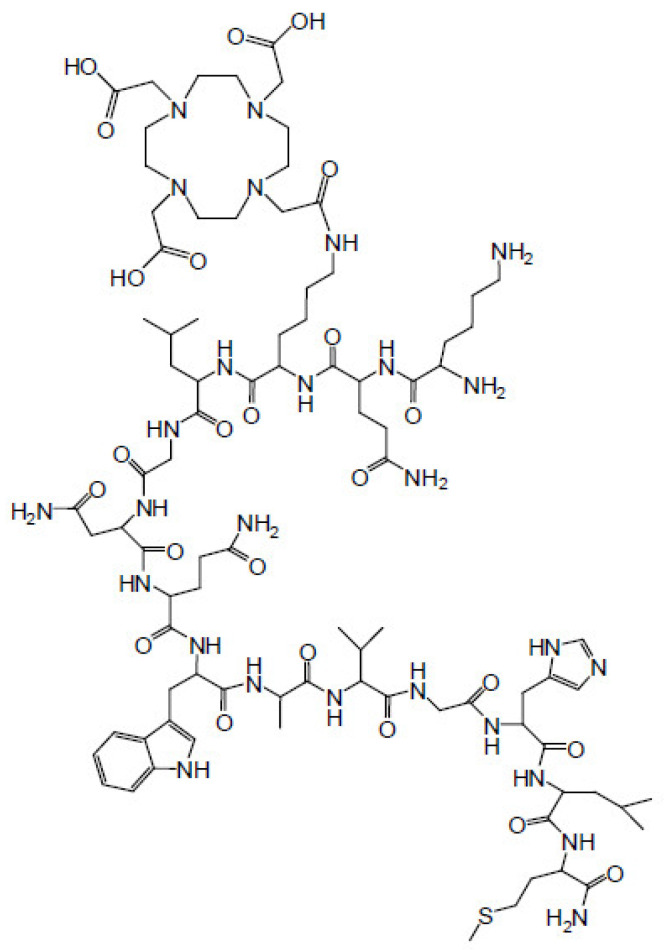
Structure of Lys1Lys3(DOTA)-bombesin 1–14.

The nanogels were modified using p-toluidine (Sigma-Aldrich) as a model compound, and with a 1,4,7,10-tetraazacyclododecane-1,4,7,10-tetraacetate (DOTA)-bombesin derivative, Lys1Lys3(DOTA)-bombesin 1-14 (BD) (Scheme 1), with the following sequence: Lys-Gln-Lys(DOTA)-Leu-Gly-Asn-Gln-Trp-Ala-Val-Gly-His-Leu-Met-NH_2_ (CS Bio Co. 95.75% and synthesized by authors). 4-(4,6-dimethoxy-1,3,5-triazin-2-yl)-4-methylmorpholinium toluene-4-sulfonate (DMT/NMM/TsO^−^) (synthesized by the Institute of Organic Chemistry, TUL) and acetonitrile for UPLC (POCH SA) were used as the coupling agent and solvent, respectively.

The step-by-step oligopeptide synthesis was performed using Rink amide resin (200–400 mesh, Sigma-Aldrich), 2-chlorotritylchloride resin (100–200 mesh, Novabiochem, Merck, Darmstadt, Germany), and protected amino acids (INTAVIS Bioanalytical Instruments AG): Fmoc-Met-OH, Fmoc-Leu-OH, Fmoc-His(Trt)-OH, Fmoc-Gly-OH, Fmoc-Val-OH, Fmoc-Ala-OH, Fmoc-Trp(Boc)-OH, Fmoc-Gln(Trt)-OH, Fmoc-Asn(Trt)-OH, Fmoc-Lys(Alloc)-OH, Fmoc-Lys(Boc)-OH, tri-tert-butyl 1,4,7,10-tetraazacyclododecane-1,4,7,10-tetraacetate (DOTA-tri-t-Bu-ester, ≥95.0%, Sigma-Aldrich), 4-methylmorpholine (NMM, ReagentPlus^®^, 99%), *N*,*N*-dimethylformamide (DMF, pure, POCH S.A.), dichloromethane (CH_2_Cl_2_, 99,8% pure P.A.-basic, POCH S.A.), piperidine (ReagentPlus^®^, 99%), methanol (MeOH, 99.8% POCH S.A.), *N*,*N*-diisopropylethylamine (DIPEA, 99.5%, Sigma-Aldrich), phenylsilane (PhSiH_3_, 97%, Fluorochem), tetrakis(triphenylphosphine)palladium(0) (Pd(PPh_3_)_4_, 99%, Sigma-Aldrich), benzotriazole-1-yl-oxy-tris-(dimethylamino)-phosphonium hexafluorophosphate (BOP, ≤100% Novabiochem), Hexafluoroisopropanol (HFIP, 99%, Sigma-Aldrich), trifluoroacetic acid (TFA, ≥99.0%, Sigma-Aldrich), 1,2-ethanedithiol (EDT, ≥98.0%, Sigma-Aldrich), and triethylsilane (TIS, 99%, Sigma-Aldrich).

Sample dialysis was performed with cellulose membrane tubing (molecular weight cut-off 14.000, Sigma-Aldrich).

The following chemicals were used for radiolabeling: L(+)-ascorbic acid (PanReac AppliChem, cat.no. 141013.1211), sodium hydroxide 30% solution (NaOH, 99%, Merck), diethylenetriaminepentaaceticacid (DTPA, Merck), and lutetium-177 (LutaPol) as lutetium chloride [^177^Lu]LuCl_3_, carrier added, of SA (specific activity) higher than 555 MBq mg^−1^ Lu in 0.04 N HCl in a volume of 0.010–2 mL, produced at Radioisotope Centre POLATOM, Otwock, Poland.

### 2.2. Radiation-Induced Synthesis of Nanogels

Poly(acrylic acid) nanogels (NG) were synthesized as reported previously [24,26] (see also Section 3.1.). Briefly, a dilute aqueous solution of linear PAA in a monomer unit concentration of 22.5 mmol · dm^−3^ (1.62 g/L) was prepared overnight by stirring at 50 °C. Since the reaction has to be performed in acidic medium, the pH of the polymer solution was adjusted to 2.0 with perchloric acid, as an acid of relatively high resistance to ionizing radiation [64]. Finally, the solution was circulated in a closed-loop system and flowed through a quartz irradiation cell, and was Ar-saturated and irradiated by short pulses of fast electrons generated by an ELU-6 linear accelerator (Elektronika, Moscow, Russia). Irradiation parameters: pulse duration 2 μs, pulse frequency 0.5 Hz, electron energy 6 MeV, absorbed dose of ionizing radiation per single pulse 0.9–1.1 kGy, as determined by alanine dosimeter (e-scan, Bruker, Billerica, MA, USA) [65]. After synthesis, the nanogel solutions were stored at 10 °C. Directly before coupling they were filtered through 1.2, 0.8, and 0.45 µm pore-size filters (Minisart NML, Sartorius, Göttingen, Germany).

### 2.3. Nanogel Coupling with a Model Compound

To examine the suitability of the chosen coupling method for the activation of the PAA nanogel carboxylic groups, first a test functionalization was performed with a simple amine model compound-p-toluidine. The coupling strategy employed was based on 4-(4,6-dimethoxy-1,3,5-triazin-2-yl)-4-methylmorpholinium toluene-4-sulfonate (DMT/NMM/TsO^−^, which will be called “ca” (coupling agent) for simplicity), the compound that was developed at the Institute of Organic Chemistry of Lodz University of Technology [52].

First, the proper amount of DMT/NMM/TsO^−^ (based on the molar ratios between the compounds in the final solution) was dissolved in ca. 5 mL of acetonitrile. Subsequently, 0.10 mmol NMM (11 μL) and 0.20 mmol NG (10 mL) were added and the solution was stirred on ice. After 40 min of carboxylic group activation, an appropriate aliquot of p-toluidine was added to the reaction. The solution was left for 12 h in an ice bath with no further ice supply to equilibrate the temperature to ambient conditions. After this time, the acetonitrile was removed from the solution using a rotary evaporator at 30 °C under vacuum. Finally, the solution was centrifuged (13 min, 9000 rpm) and the precipitate (unreacted substrates) was removed. According to the abovementioned procedure, four different reactions with varying molar ratios between carboxylate groups of PAA nanogels (NG), DMT/NMM/TsO- (ca), and p-toluidine molecules (T) were carried out as specified in Table 1. The samples after coupling were stored at a temperature of 10 °C.

### 2.4. In-House Synthesis of Custom Bombesin Derivative

To prove the versatility of the chosen *N*-triazinylammonium sulfonate coupling strategy, synthesis of the DOTA-bombesin derivative was also attempted in our lab. The peptide was built from the Fmoc-protected amino acids using solid-phase peptide synthesis (SPPS) and 4-(4,6-dimethoxy-1,3,5-triazin-2-yl)-4-methylmorpholinium toluene-4-sulfonate as a condensing reagent. The stepwise synthetic procedure is described below.

#### 2.4.1. Rink Amide Resin Loading

First, amino acid–Fmoc-protected methionine (3 equiv. rel. to the 0.40 mmol loaded resin = 1.20 mmol), DMT/NMM/TsO^−^ (3 equiv. rel. to the resin = 1.20 mmol), and NMM (6 equiv. rel. to the resin = 2.40 mmol) were dissolved in DMF (4 mL). A total of 400 mg of Rink amide resin (amine group load of 1.00 mmol/g) was preswollen in 3 mL of CH_2_Cl_2_ for 1 h, and after this time the solution containing the protected amino acid was added and the resin was shaken for 24 h. Finally, the resin was washed with 4 mL DMF (3×) and 4 mL CH_2_Cl_2_ (3×). A Kaiser test [66] confirmed complete resin loading.

#### 2.4.2. Deprotection

The Fmoc protecting group was removed by treatment with a 4 mL solution of 25% piperidine in DMF (2 × 15 min). After deprotection, the resin was washed with 4 mL of DMF (3×) and 4 mL of CH_2_Cl_2_ (3×).

#### 2.4.3. Standard Coupling Procedure

Protected amino acid (3 equiv. rel. to the resin = 1.20 mmol), DMT/NMM/TsO^−^ (3 equiv. rel. to the resin = 1.20 mmol), and NMM (6 equiv. rel. to the resin = 2.40 mmol—approx. 300 µL) were dissolved in DMF (4 mL) and added to the resin. The resin was shaken for 24 h. The progress of the reaction was monitored by the Kaiser test [66].

The following amino acids were coupled in a growing peptide chain by stepwise addition: Fmoc-Met-OH, Fmoc-Leu-OH, Fmoc-His(Trt)-OH, Fmoc-Gly-OH, Fmoc-Val-OH, Fmoc-Ala-OH, Fmoc-Trp(Boc)-OH, Fmoc-Gln(Trt)-OH, Fmoc-Asn(Trt)-OH, Fmoc-Gly-OH, and Fmoc-Leu-OH. The last seven amino acids were coupled using a LibertyBlue device (CEM Company, Matthews, NC, USA) that generates microwaves, accelerating the coupling process. The resin was dried after the coupling procedure.

#### 2.4.4. 2-Chlorotrityl Chloride Resin Loading

Protected amino acid-Fmoc-Lys(Alloc)-OH (3 equiv. rel. to the resin = 1.11 mmol) and DIPEA (6 equiv. = 2.22 mmol approx. 400 µL) were dissolved in CH_2_Cl_2_ (10 mL per 1 g of the resin). A total of 300 mg of 2-chlorotritylchloride resin (load carboxylic groups 1.23 mmol/g) was preswollen in 3 mL CH_2_Cl_2_ for 1 h. After this time, the solution containing the protected amino acid was added and the resin was shaken for 2 h. Finally, the resin was washed with CH_2_Cl_2_/MeOH/DIPEA (17:2:1, 20 mL), then 4 mL DMF (2×) and 4 mL CH_2_Cl_2_ (3×).

#### 2.4.5. Deprotection

To remove the Alloc protecting group of the lysine, PhSiH_3_ (24 equiv. rel. to the resin = 4.82 mmol) in 2 mL of CH_2_Cl_2_ in an atmosphere of inert gas (nitrogen) was added. Subsequently, after two minutes, 0.25 equiv. rel. to the resin = 0.05 mmol Pd(PPh_3_)_4_ in 6 mL of CH_2_Cl_2_ was added and the resin was shaken for 40 min. As a last step, the resin was washed with 3 mL CH_2_Cl_2_ (3×), 3 mL DMF (3×), and 3 mL CH_2_Cl_2_ (4×). The progress of the reaction was monitored by the Kaiser test [66].

#### 2.4.6. Coupling of Tri-tert-butyl 1,4,7,10-tetraazacyclododecane-1,4,7,10-tetraacetate to Unprotected Lysine

DOTA-tri-t-Bu-ester (1.70 equiv. rel. to the resin = 0.35 mmol), DMT/NMM/TsO^−^ (1.70 equiv. rel. to the resin = 0.35 mmol), and NMM (4 equiv. rel. to the resin = 0.80 mmol) were dissolved in CH_2_Cl_2_/DMF (1:1, 6 mL), added to the resin, and shaken for 12 h. Then, the solution was removed and the resin was washed with CH_2_Cl_2_. The procedure, with the addition of DOTA-tri-t-Bu-ester, was repeated. The resin was again shaken in the mixture for 12 h. The resin grains remained purple after the Kaiser test, so coupling was repeated again, but with a different coupling agent—BOP. DOTA-tri-t-Bu-ester (1.7 equiv. rel. to the resin = 0.349 mmol), BOP (1.7 equiv. rel. to the resin = 0.349 mmol), and NMM (over 4 equiv. rel. to the resin, exactly 0.9 mmol) were dissolved in CH_2_Cl_2_/DMF (1:1, 4 mL) and added to the resin. The resin was shaken for 24 h.

#### 2.4.7. Cleavage from the 2-Chlorotritylchloride Resin

Fmoc-Lys(DOTA-tri-t-Bu-ester)-OH was cleaved from the resin using HFIP/DCM (1:1, 20 mL). Cleavage was carried out for 3.5 h, then the resin was filtered off and the filtrate was evaporated under a nitrogen flow. The resulting precipitate was dissolved in deionized water/acetonitrile (1:1, 6 mL) and then frozen in liquid nitrogen and lyophilized.

#### 2.4.8. Coupling of Fmoc-Lys(DOTA-tri-t-Bu-ester)-OH to Peptide on Rink Amide Resin

Fmoc-Lys(DOTA-tri-t-Bu-ester)-OH was coupled using the LibertyBlue device. The Rink amide resin was preswollen in 3 mL CH_2_Cl_2_ for 1 h. A solution with a concentration of 0.50 mol · dm^−3^ of DMT/NMM/TsO^−^ in DMF and 2.00 mol · dm^−3^ solutions of NMM in DMF were prepared. Initially, from the last amino acid on the Rink amide resin—leucine—the Fmoc group was removed by treatment with a solution of 25% piperidine in DMF. Then, DMT/NMM/TsO^−^ solution was added to the peptide, as well as Fmoc-Lys(DOTA-tri-t-Bu-ester)-OH solution in DMF and NMM solution. At the end of the coupling, the resin was washed with DMF and then dried.

#### 2.4.9. Coupling of Fmoc-Gln(Trt)-OH and Fmoc-Lys(Boc)-OH

The last two amino acids were coupled using the LibertyBlue device. The Rink amide resin was preswollen in CH_2_Cl_2_ for 1 h. A total of 0.50 mol · dm^−3^ solution of DMT/NMM/TsO^−^ in DMF and 2.00 mol · dm^−3^ solution of NMM in DMF was prepared. First, from the last amino acid on the Rink amide resin–lysine—the Fmoc group was removed by treatment with a solution of 25% piperidine in DMF. Then, DMT/NMM/TsO^−^ solution was added to the peptide and Fmoc-Gln(Trt)-OH solution in DMF (0.20 mol · dm^−3^) and NMM solution. Then, the resin was washed with DMF and deprotection and coupling was repeated for Fmoc-Lys(Boc)-OH. The resin was washed with DMF and then dried.

#### 2.4.10. Cleavage from the Rink Amide Resin

The peptides were cleaved from the resin using TFA/H_2_O/EDT/TIS (94%, 2.5%, 2.5%, 1%, 5 mL) solution. Cleavage was carried out for 4 h, then the resin was filtered off and the filtrate was evaporated under a nitrogen flow. The resulting precipitate was dissolved in deionized water/acetonitrile (1:1, 6 mL) and then frozen in liquid nitrogen and lyophilized.

#### 2.4.11. Synthesis of Bombesin Derivative without DOTA

As a reference material, a DOTA-free bombesin derivative was synthesized in a process similar to the abovementioned procedure. Fmoc-protected amino acids were coupled with DMT/NMM/TsO^−^ and NMM on Rink amide resin. The protecting groups were removed by treatment with a solution of 4 mL 25% piperidine in DMF (2 × 15 min), and after deprotection, the resin was washed with 3 mL DMF (3×) and 3 mL CH_2_Cl_2_ (3×).

### 2.5. Nanogel Coupling with Bombesin Derivative

The method developed by Kamiński and Kolesińska, described above for the model compound, was repeated for two types of bombesin derivatives (bombesin derivative without DOTA—in-house synthesized; DOTA-bombesin derivative, both in-house synthesized and later supplied externally by CS Bio Co.). DMT/NMM/TsO^−^ was dissolved in ca. 5 cm^3^ acetonitrile and then 11 μL of NMM (0.10 mmol) and 10 mL of PAA nanogel solution (0.20 mmol) were added. The molar ratio of the coupling agent to PAA nanogel carboxylic groups was 1:1 for the activation of all carboxylic groups. The samples after completed coupling were stored at 10 °C. Aliquots of both nanocarriers and raw nanogels were freeze-dried, (e.g., for radiolabeling experiments) and stored at −20 °C.

Coupling was performed with three different molar ratios of carboxylic groups of PAA nanogel to bombesin derivative, as described in detail in Table 2.

### 2.6. Purification of Nanogels Coupled with Model Compound/Oligopeptide

Low-molecular-weight compounds formed during the coupling process (such as 2-hydroxy-4,6-dimethoxy-1,3,5-triazine and *N-*methylmorpholine p-toluenesulfonate), as well as unreacted reagents, were removed by dialysis in cellulose membrane tubing (molecular weight cut-off 14.000, Sigma-Aldrich) against a 30-fold excess of deionized water at room temperature. The purification process was controlled by absorbance measurements. Wherever the properties of coupled nanogels were compared to the raw nanoparticles, solutions of the latter were subjected to the same dialysis procedure before measurements.

### 2.7. Characterization of Nanogels

#### 2.7.1. Static Light-Scattering Measurements (SLS)

The weight-average molecular weight and radius of gyration of linear PAA and PAA nanogels were determined by static multiangle laser light-scattering measurements using a BI-200SM goniometer (Brookhaven Instruments Corporation, Holtsville, NY, USA) with an Innova 90C Ar ion laser (*λ* = 514.5 nm) at 25.0 ± 0.1 °C, in an aqueous solution of 0.5 mol · dm^−3^ NaClO_4_, pH 10 (NaOH). This solvent ensured a compact conformation of PAA macromolecules required for the light-scattering measurements. The Zimm algorithm was used to analyze the SLS data, assuming the refractive index increment d*n*/d*c* = 0.30 cm^3^ g^−1^ [67]. Linear PAA and PAA nanogel solutions were filtered through a 0.45 μm-pore-size filter (Minisart NML, Sartorius) before measurement, and the solvent was filtered through a 0.2 μm-pore-size filter (Minisart NML, Sartorius).

#### 2.7.2. Dynamic Light-Scattering Measurements (DLS)

To supplement the SLS data, the hydrodynamic radii of linear PAA and PAA nanogels in an aqueous solution of 0.5 mol · dm^−3^ NaClO_4_, pH 10 (NaOH), were measured using ZetaSizer Nano ZS (Malvern Instruments Ltd., Malvern, Worcestershire, United Kingdom) equipped with a 633 nm laser (25.0 ± 0.1 °C). The purified nanogels, both raw and coupled with model compound/oligopeptide, were measured with the same device at neutral pH at 25.0 ± 0.1 °C.

#### 2.7.3. FTIR Infrared Spectroscopy

FTIR spectra of PAA nanogels, both before after coupling with the model compound or oligopeptide, were recorded in the transmission mode using a Nicolet Avatar 330 FTIR spectrophotometer (Thermo Nicolet Corporation, Madison, WI, USA) in the range of 400 to 4000 cm^−1^. Before measurement, the samples were freeze-dried, then mixed with KBr in a 1:100 *w*/*w* ratio and formed into pellets.

#### 2.7.4. UV-Vis Spectroscopy

UV-Vis spectra were recorded to determine the efficiency of the product purification by dialysis. This method was also used to track the coupling of the model compound and oligopeptide to the nanogels. UV-Vis measurements in quartz cells of a 1 cm optical path were performed against water in the wavelength range of 190–900 nm using a Lambda 40 double-beam spectrophotometer (Perkin-Elmer, Waltham, MA, USA).

#### 2.7.5. Fluorescence Spectroscopy

Emission spectra of raw PAA nanogels, bombesin-DOTA derivative, and PAA nanocarriers coupled with bombesin-DOTA derivative were collected using an Aminco-Bowman Series 2 spectrofluorometer (Spectronic Unicam, Rochester, NY, USA) equipped with a xenon lamp and a red-sensitive photomultiplier (Hamamatsu R928). The excitation wavelength was 280 nm for the excitation of tryptophan present in the oligopeptide structure. The excitation and emission slits were set to 4.0 and 2.0  nm, respectively. The fluorescence measurements were performed in quartz cells with a 1 cm optical path.

#### 2.7.6. ^1^H NMR Nuclear Magnetic Resonance Spectroscopy

^1^H NMR spectra were recorded for the nanogels before and after coupling with the model compound/oligopeptide. The freeze-dried samples were dissolved in deuterated water. The measurements were performed using a Bruker Avance II 700 MHz UltraShield Plus NMR spectrometer (Bruker, Billerica, MA, USA).

### 2.8. Radiolabeling of DOTA-Bombesin Derivative and Quality Control

The samples of PAA nanogels coupled with DOTA-bombesin derivative were labeled with [^177^Lu]LuCl_3_ (0.2–9.8 GBq). Each of the 1 mg lyophilized NGBD samples was dissolved in 1 mL of water for HPLC (high-performance liquid chromatography). Next, 0.5 mL of NGBD was mixed with 0.2 mL of ascorbic acid sodium salt solution of pH = 4.5–5.0 and 2–100 µL of radionuclide were added. The sample was incubated at 95 ± 5 °C for 15 min.

The final formulation’s radiochemical yield (RCY) was determined by thin-layer chromatography on glass-fiber silica gel-coated plates (ITLC SG) with 0.2 M potassium chloride pH = 2.0–2.5 as a mobile phase to differentiate between the free radionuclide and radiolabeled PAA nanogels. The radiolabeling yield was evaluated in a competitor’s presence (10 mM DTPA) in excess, reacting with the unbound radionuclide.

## 3. Results and Discussion

### 3.1. Radiation-Induced Synthesis of Nanogels

Radiation-induced nanogel synthesis seems to be a convenient tool for obtaining carriers for oligopeptides, radioactive substances, or genes. This process is carried out without potentially harmful compounds, like catalysts, initiators, etc., which are vital in chemical synthesis. The mechanism of radiation-induced nanogel synthesis from dilute aqueous polymer solutions is well known [21,23,24,25,68,69,70,71,72]; ionizing radiation (e.g., γ-rays or electron beam) is absorbed by water and as a result, short-lived reactive species as hydroxyl radicals, hydrated electrons, and hydrogen atoms are formed. To maximize the yield of these radicals and avoid the formation of peroxyl radicals and subsequent polymer degradation, the solutions are saturated with argon or nitrous oxide [64,67,73,74]. The next step in the deoxygenated solution is hydrogen atom abstraction from macromolecules by ●OH and H●, resulting in the formation of radicals on polymer chains. These radicals recombine with each other inside the polymeric coil, leading to intramolecular crosslinking and the formation of nanogels. PAA is a weak polyelectrolyte, so for nanogel synthesis, the appropriate pH is required where the chains attain coiled conformation and their segments do not repel each other. In a strongly acidic environment, below pH 2, PAA chains form hydrogen bonds and aggregate. At pH 2, which is used during synthesis, carboxylic groups are protonated and there is less than 1% of charged units; therefore, the polymer behaves nearly like a neutral chain [75]. In more alkaline and neutral pH, above the p*Ka* of ca. 6.0–6.2 [76] the dominant reaction during the irradiation of PAA solutions is chain scission due to Coulombic forces between groups with the same electrostatic negative charge COO^−^, which keep the chain segments apart and thus prevent crosslinking. In the conditions exploited in this procedure, the predominant reaction was intramolecular crosslinking, which led to nanogel synthesis.

In this paper, PAA nanogels were synthesized by the above-described method [26], with the aim of their subsequent modification by conjugation, first by a simple model compound and finally by the oligopeptide—bombesin. This section briefly describes the synthesis and physical properties of PAA nanogels before coupling. The weight-average molecular weight (*M_w_*) and dimensions of the radius of gyration (*R_g_*) and hydrodynamic radius (*R_h_*), as well as the density of the polymer coil (*ρ_coil_*), are shown in Table 3 for two 22.5 mol · dm^−3^ solutions that were irradiated during two separate accelerator sessions, yielding two batches of nanogels. As a reference, the results obtained for the pristine linear polymer are also shown in the table. It should be noted that these parameters were measured under specific conditions, namely, in an aqueous solution of 0.5 mol · dm^−3^ NaClO_4_, pH 10. This solvent ensured a compact conformation of PAA macromolecules required for the light-scattering measurements.

**Figure 1:**
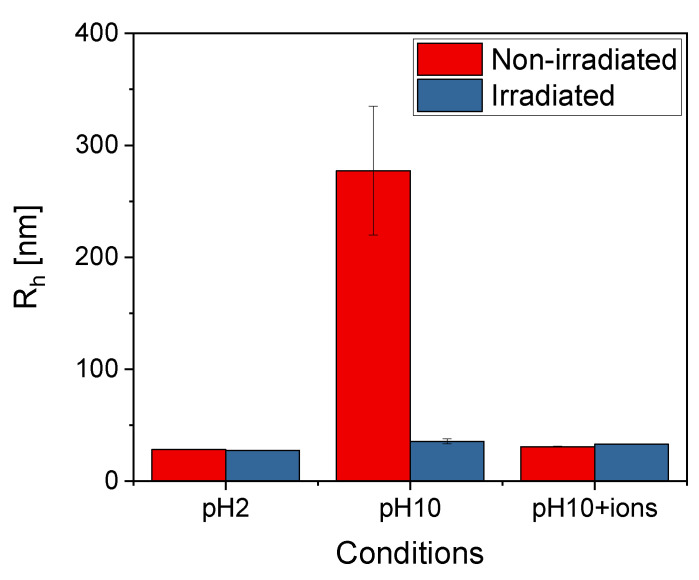
Hydrodynamic radii of PAA nanogels in aqueous solution at various conditions. pH 10+ions indicates aqueous solution containing 0.5 mol · dm^−3^ NaClO_4_, pH 10.

The weight-average molecular weight of the pristine polymer in the solution (pH = 10; 0.5 mol · dm^−3^ NaClO_4_) was found to be 8.87 × 10^5^ Da, with radius of gyration of 115 nm and a hydrodynamic radius of 42 nm. Two batches of nanogels were synthesized by pulse-irradiating deoxygenated aqueous solutions of PAA at pH 2 with a dose of ca. 5 kGy. The nanogels from the first batch, marked as NG_I (dose 5.3 kGy, final *M_w_* = (1.26 ± 0.09) × 10^6^ Da, *R_g_* = 94 ± 5 nm, and *R_h_* = 45 ± 4 nm), were used, without further filtration, for coupling with the model compound and DOTA-bombesin derivative supplied by an external company. The nanogels of the second batch NG_II (dose 5.3 kGy, final *M_w_* = (1.13 ± 0.11) × 10^6^ Da, *R_g_* = 86 ± 22 nm, and *R_h_* = 35 ± 0.8 nm), prior to further coupling steps, were filtered and the parameters changed to *M_w_* = (3.83 ± 0.3) × 10^5^ Da, *R_g_* = 41 ± 2 nm, and *R_h_* = 30 ± 4 nm. These nanogels were used for coupling tests with DOTA-bombesin derivative and bombesin derivative without DOTA, which were synthesized in the authors’ laboratory. The absorbed dose of ca. 5 kGy was chosen to induce sufficient internal crosslink density and stability of the so-formed structures. It was found that they were colloidally stable, both in water and biologically relevant solvents such as PBS or cell culture media (RMPI1640). Moreover, they could be also safely freeze-dried or stored for at least two months (data not shown). The density of the polymer coil of NG_I and NG_II was much higher than the original coils of the linear polymer. This result confirms that intramolecular crosslinking plays a dominant role during radiation synthesis, and as a result, more densely packed polymer coils are obtained; this phenomenon is characteristic for nanogels as internally crosslinked macromolecules [77]. To further prove this, the hydrodynamic radius was also measured at various pH and ion strength conditions (pH 2 vs. pH 10 vs. aqueous solution of 0.5 mol · dm^−3^ NaClO_4_, pH 10) (Figure 1). It can be seen that in contrast to the non-irradiated linear polymer, the irradiated samples retained the compact conformation and small size in the changing environment.

### 3.2. Coupling PAA Nanogels with a Simple Amine as Model Compound for Oligopeptide

As a model compound for the oligopeptide, a simple aromatic amine-p-toluidine was used. PAA nanogels were successfully conjugated with p-toluidine using 4-(4,6-dimethoxy-1,3,5-triazin-2-yl)-4-methylmorpholinium toluene-4-sulfonate (DMT/NMM/TsO^−^), which was developed by Kamiński and Kolesińska [51,52]. This salt activates carboxylic groups, which in turn could easily react with the amines in the model compound or oligopeptide. Various reagent ratios were tested (see Table 1 in Section 2.3). The samples after coupling were centrifuged and the precipitate was removed. The obtained products, especially NG/ca/T 1/1/1, were cloudy; therefore, all the samples were centrifuged. All four coupling products were dialyzed until low-molecular-weight products were removed.

After dialysis, the sizes (*R_h_*) of the model nanocarriers were measured (Table 4) using the DLS technique in water at pH ≈ 4. It should be noted that these conditions were different from those used to determine nanogel parameters just after their synthesis (dialyzed samples measured in water instead of non-dialyzed ones in a solution of 0.5 mol · dm^−3^ NaClO_4_, pH 10); hence, the size of the non-modified nanogels, NG_I, differed from that listed in Table 3. The average *R_h_* values for all samples were above 100 nm. It is interesting to see that toluidine coupling resulted in a decrease in the nanogel size. This can be interpreted as the result of partial hydrophobization of the initially highly hydrophilic gel particle, which was strongly swollen in water. Hydrophobic interactions between the toluidine groups within the nanogels apparently caused the structure to shrink.

Physicochemical properties of purified nanogels coupled with p-toluidine were analyzed by FTIR, UV-Vis, and ^1^H NMR spectroscopy to confirm successful coupling.

**Figure 2:**
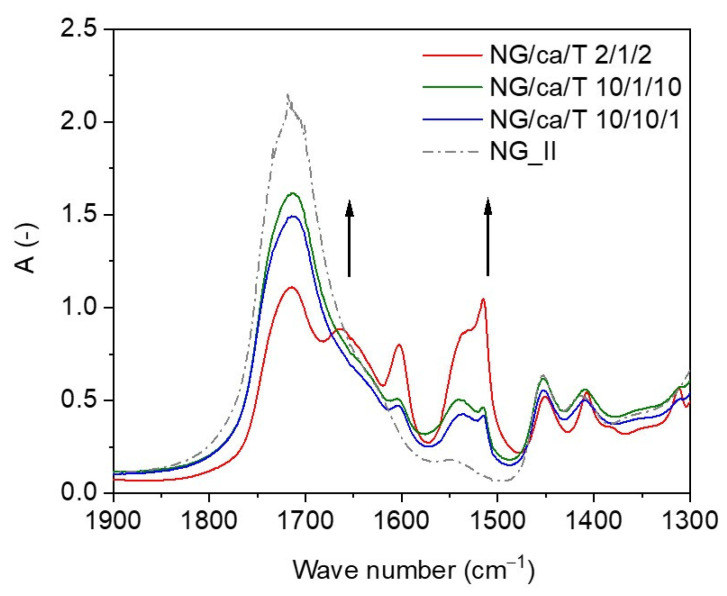
FTIR spectrum of three products of PAA nanogels coupling with p-toluidine (NG/ca/T) in different molar ratios: 2/1/2 and 10/10/1, and neat poly(acrylic acid) nanogels (NG_II).

Nanocarriers with the model compound were analyzed by FTIR spectroscopy; the spectra of three products of PAA nanogels coupled with p-toluidine were compared with PAA nanogels after dialysis and lyophilization (Figure 2). In the literature, the spectra of PAA [78,79,80] and p-toluidine [81,82,83] are well known. The PAA spectrum was characterized by a broad band at ~3140–3190 cm^−1^ corresponding to O‒H stretching, which overlapped with a C‒H stretching band near 2960 cm^−1^ and a separate strong band 1712 cm^−1^ from C=O stretching. Many other smaller bands could be also found: at 1454 cm^−1^ derived from CH_2_ deformation vibrations; three bands near 1415 cm^−1^, 1246 cm^−1^, and 1171 cm^−1^ that corresponded to C‒O stretching vibrations coupled with O‒H in-plane bending; a broad band at 902 cm^−1^ from O‒H out-of-plane bending; and 801 cm^−1^ from CH_2_ twisting and C‒COOH stretching. The spectra of coupling products were similar to those of raw PAA nanogels; however, one can easily distinguish a broad double band near 1515 and 1540 cm^−1^ that originated from amide II derived mainly from in-plane N-H bending and to some extent from C‒N and C‒C stretching vibrations, and probably from p-toluidine C=C stretching and NH_2_ bending vibrations (1510, 1594, and 1625 cm^−1^). The highest intensity of these signals could be observed for the sample NG/ca/T 2/1/2, whereas it was significantly weaker for the samples of molar ratios 10/1/10 and 10/10/1. Another characteristic band expected for p-toluidine coupled with nanogels, at ca. 1663 cm^−1^, was apparently overlapped by the strong C=O stretching band of PAA. A closer look at the strong maximum for PAA at 1712 cm^−1^ reveals that it became wider upon coupling, suggesting some contribution from the C=O stretching band of the amide.

**Figure 3:**
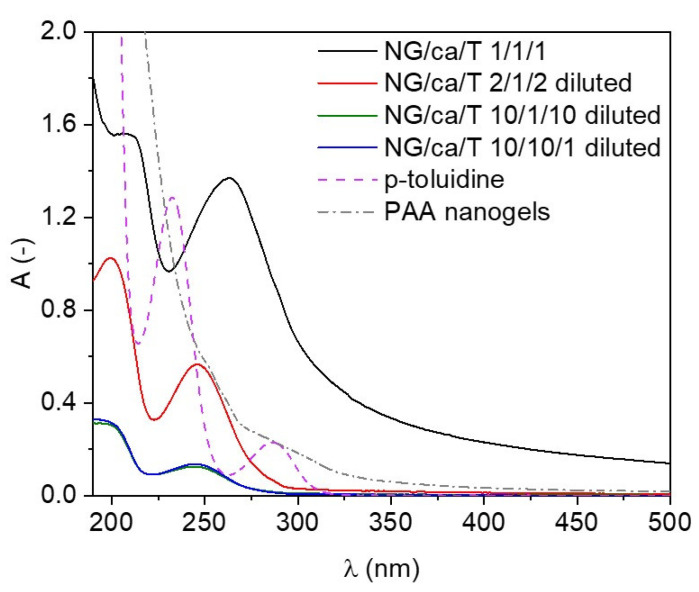
The UV-Vis spectra of four product samples of PAA nanogel coupling with p-toluidine (NG/ca/T) in different molar ratios: 1/1/1 (pH 4.3), 2/1/2, 10/1/10, and 10/10/1; the three last samples were diluted 100× and their pH was ca. 6. The pink dashed line corresponds to 0.16 mmol · dm^−3^ p-toluidine solution, pH 5.5; the grey dotted line corresponds to PAA nanogels after dialysis, pH 5.7.

The coupling process was also followed by UV-Vis spectroscopy. The spectra of 0.16 mmol · dm^−3^ aqueous p-toluidine solution, pH 5.5, showed three bands at 198, 232, and 287 nm (Figure 3) [84], whereas neat PAA nanogels showed a featureless spectrum with absorbance rising towards the far UV. These two spectra were compared with data obtained for products of the coupling process. Bands for the coupled products were visible in range of 190–325 nm, with the maximum at 198 nm and 245 nm, which was shifted to 260 nm for NG/ca/T 1/1/1 sample (Figure 3). Comparing the spectrum of p-toluidine and the coupling products, one can see a certain shift in the bands. The pH of all samples was set as a value above the p-toluidine p*K_a_* = 5.1 [85]; hence, these differences did not originate from varying pH values, but rather from differences in the electronic structure between the starting amine and resulting amide, which may have been particularly pronounced in this system due to the aromatic character of the substituent. The peak shifts in the coupling products spectra made it impossible to quantify the content of p-toluidine. Therefore, another method was used to characterize the product samples—^1^H NMR spectroscopy.

**Figure 4:**
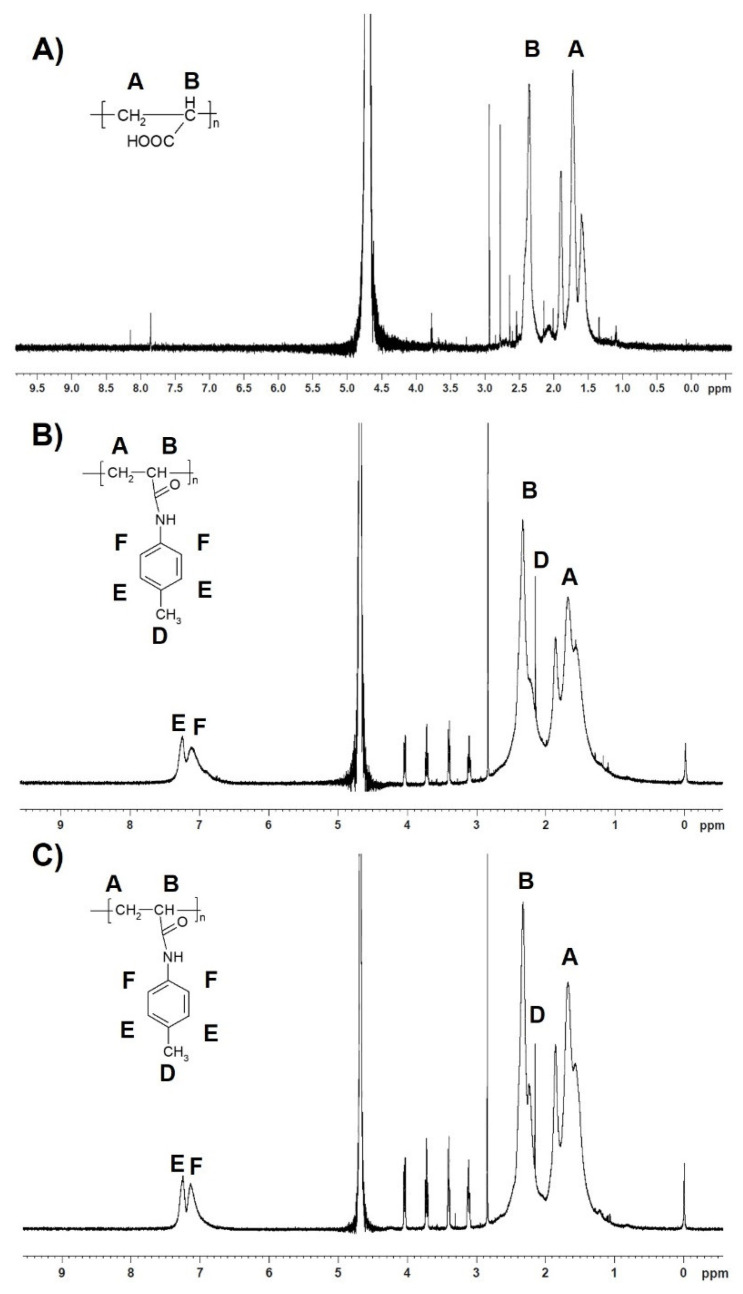
^1^H NMR spectra of (**A**) poly(acrylic acid) nanogels, (**B**) nanogels of PAA/coupling agent/p-toluidine in molar ratio 10/10/1, and (**C**) PAA/coupling agent/p-toluidine in a molar ratio of 10/1/10 recorded in D_2_O, 700 MHz.

The ^1^H NMR spectrum of linear PAA has been studied before [86,87,88]. The experimental spectrum of the PAA nanogels (Figure 4A) is consistent with those in the literature. High-intensity signal A (1.7 ppm) corresponded to the group CH_2_ and B (2.3 ppm) of the CH protons of poly(acrylic acid) nanogels. The integration of peaks, as in the literature spectrum, gave integral ratios for the poly(acrylic acid) nanogel backbone H_A_:H_B_ = 2:1. Other bands of low intensity visible in the spectrum may have been a result of polymer chain fragmentation during irradiation, but the bands were so small that they were considered insignificant. The large peak at 4.7 ppm came from water and was visible in each spectrum.

The spectra of the substituted nanogels (PAA nanogels/coupling agent/p-toluidine in molar ratio 10/10/1 and 10/1/10, Figure 4B,C, respectively) were compared to the neat nanogel spectrum. Both coupling product spectra were very similar to each other. In the spectrum from 1 to 3 ppm, several signals came from poly(acrylic acid), doublet (d) of the -CH_2_ group (A; 1.5 ppm), and triplet of the -CH group (B, 2.3 ppm). The signals from the aromatic group of p-toluidine (available in the database, recorded in D_2_O, 700 MHz) [89,90,91,92] were visible—two doublets E (7.2 ppm) and F (7.1 ppm) and singlet (s) of the -CH_3_ group D (2.1 ppm). Although exact quantitative analysis was not possible due to the broadening and overlap effects, a rough estimate based on integrating peaks A, B, and D, as well as E and F, was that for 10/10/1 and 10/1/10, ca. 15 mol% and 12 mol% of the original carboxylic groups were substituted, not very far from the theoretical yield of ca. 10%. This seems to indicate that the theoretical stoichiometric degree of substitution was achieved and points to somewhat higher coupling efficiency in the case of 10/10/1, in line with the FTIR data in Figure 2.

The peaks in the range of 3–4 ppm visible in the spectra in Figure 4B,C were derived from *N*-methylmorpholine (NMM), which was added at the stage of activation of the carboxylic function in order to convert the COOH group into a carboxylate anion (salt from NMM), which, due to its higher reactivity, is easier to react with condensing reagents to form active forms.

To summarize, the tests on the coupling model compound with PAA nanogels confirmed the effectiveness of described coupling method. The studies show that the molar ratio of PAA nanogels/coupling agent/p-toluidine 1/1/1 is too large. The 2/1/2 sample also had some precipitation issues, but the UV-Vis and FTIR measurements confirmed that the coupling process was successful. The sample 10/10/1 had the best efficiency in comparison to other samples, with no precipitation effects observed. Somewhat less of the model compound was covalently bound in the sample of PAA nanogels/coupling agent/p-toluidine 10/1/10. Such results were expected, because in the last case 10-fold less carboxyl groups were activated than in the case of sample 10/10/1. This is an important indication for further steps in bombesin coupling. Each time the coupling agent was used to activate all carboxylic groups on the nanogels, and only the COOH-oligopeptide ratio was changed.

### 3.3. Coupling PAA Nanogels with Oligopeptide

The next step after coupling the PAA nanogels with the model compound was coupling the nanogels with oligopeptide—bombesin derivative. The studies were performed on two types of oligopeptide (with and without DOTA), originating from two sources: one in-house synthesized (both variants of peptide) and the second supplied by an external company (DOTA-bombesin derivative). All bombesin derivatives were coupled with PAA nanogels using 4-(4,6-dimethoxy-1,3,5-triazin-2-yl)-4-methylmorpholinium toluene-4-sulfonate. The experience gained from preliminary studies on the model compound has shown that the best option is the activation of all carboxylic groups of PAA nanogels, so the molar ratio of the nanogels and coupling agent during reaction was fixed. The molar ratio of the carboxylic groups of PAA nanogels to oligopeptide had to be high enough not to change hydrophilic properties of the nanocarriers. Coupling was performed using three different molar ratios of carboxylic groups in PAA nanogels to bombesin derivative: 100/1 (for in-house synthesized—NGBD100H and supplied by external company, DOTA-bombesin derivative—NGBD100E, and one sample without DOTA—NGB100H), 500/1—NGBD500E, and 1000/1—NGBD1000E for the samples of DOTA-bombesin derivative supplied by an external company. The first step in the coupling reaction is the activation of carboxylic groups of PAA nanogels by adding DMT/NMM/TsO^−^ and the next step is the final coupling of the oligopeptide with nanogel. 

The measurements of dynamic light scattering were performed on the purified samples of nanogels coupled with bombesin—DOTA derivative (pH 6.3). Table 5 summarizes the average values of the Z-average hydrodynamic radius. The *R_h_* values of the substituted products did not differ significantly from the hydrodynamic radii of their parent nanogels NG_I and NG_II for the “E” and “H” series, respectively. This could have been due to the relatively low degree of substitution of the carboxylic groups (maximum one bombesin molecule per 100 groups), but also to the two counterbalancing effects of substitution–increase in the mass (which could have manifested itself as a larger size if all other factors remained unchanged) and decrease in hydrophilicity, which in turn may have caused some de-swelling of the nanogels.

**Figure 5:**
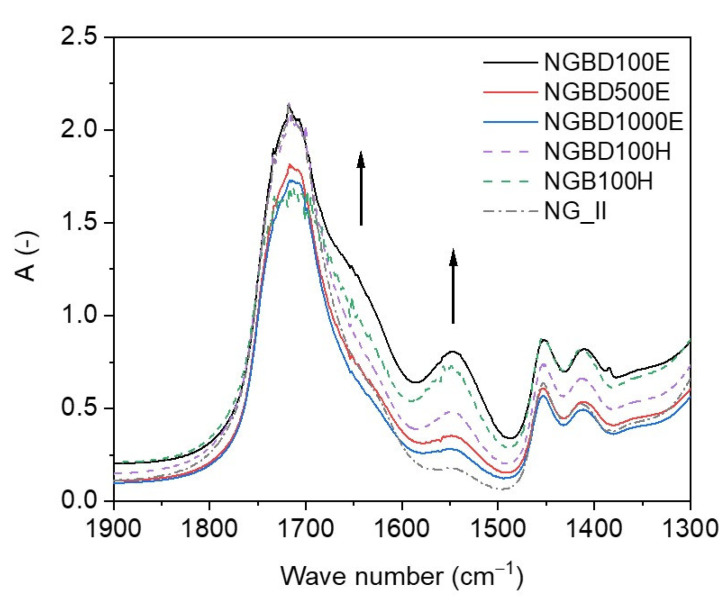
FTIR spectra of neat and bombesin-substituted PAA nanogels NG_II (sample codes—see Table 2).

In order to confirm the coupling of the nanogels to the bombesin derivative, FTIR, UV-Vis, fluorescence, and ^1^H NMR spectroscopy were employed. The FTIR spectrum of the PAA nanogels was discussed in Section 3.2, and in Figure 5 it is compared with all the coupling products. According to the literature, bombesin-DOTA derivative has two characteristic bands: at 3299 cm^−1^ free amine groups, at 1647 cm^−1^ carbonyl groups of the peptide bonds (amide I), and at 1533 cm^−1^ CN (amide II) [93,94]. The band of bombesin-DOTA derivative amine groups was overlayed by O‒H stretching and C‒H stretching PAA bands. The strong maximum at 1712 cm^−1^ from the C=O stretching bands of the carboxylic groups of the nanogels overlapped with the carbonyl groups of the peptide bond and amide I band near 1665 cm^−1^. In this region, the signal from the carbonyl groups of DOTA should also have been visible at 1734 cm^−1^ [95]. The only characteristic feature of the peptide that was not overlayed by the PAA signals was a band at 1547 cm^−1^, derived from amide II, representing the amide group formed by coupling the nanogels with the oligopeptide and bombesin-DOTA derivative CN groups. The intensity of the amide II bands for the spectra of the PAA nanogels coupled with bombesin-DOTA derivative in the ratio of 100/1 (samples NGBD100E, NGBD100H, Figure 5) was the highest, and for these samples the band near 1712 cm^−1^ was also wider than for PAA, confirming the presence of amide I. The intensity of these bands was much lower for the samples with significantly smaller amounts of bombesin-DOTA derivative introduced into the reaction (Figure 5).

**Figure 6:**
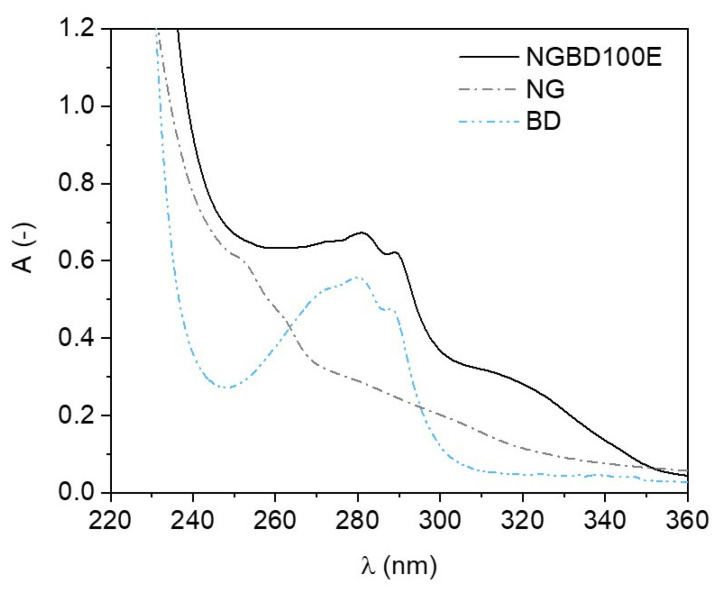
UV-Vis spectra of the substrates—0.367 gl · L^−1^ DOTA-bombesin derivative (BD), raw purified PAA nanogels after dialysis, pH 3.8 (NG), and the coupling product—nanocarrier obtained from coupling PAA nanogels with DOTA-bombesin derivative in a molar ratio of 100/1 (NGBD100E), pH 6.3.

In Figure 6, the UV-Vis spectrum of a representative sample—a nanocarrier obtained by coupling PAA nanogels with DOTA-bombesin derivative in the molar ratio of 100/1 (NGBD100E)—is compared to the spectra of the substrates: DOTA-bombesin derivative and raw purified PAA nanogels (pH 3.8, concentration after dialysis ca. 1.01 gl · L^−1^). Although the nanogel spectrum was broad and featureless, the DOTA-bombesin exhibited three characteristic overlapping bands at 273 nm, 280 nm, and 289 nm, in agreement with the literature data [94]. These bands were also present in the spectra of nanogels coupled with bombesin-DOTA. Based on the absorbance at 280 nm after subtraction of the absorbance of pure nanogels, one can estimate the content of bombesin-DOTA in the products (Table 6). Although this estimate is not a precise one (which can be seen from one of the results exceeding 100%), some conclusions can be drawn. In general, the coupling efficiencies were reasonably high, especially taking into account the fact that some carboxylic groups in the nanogel structure might have been less easy to reach or even less sterically suitable for coupling. On average, somewhat higher coupling efficiency was reached for samples where relatively high amounts of bombesin was used in the coupling reaction, whereas for the NGBD1000E sample (lowest amount of bombesin) the lowest value was observed.

**Figure 7:**
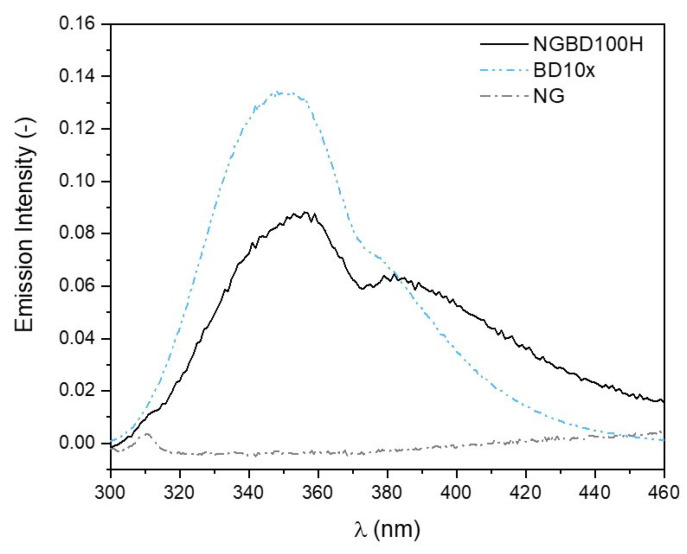
Emission fluorescence spectra of the substrates—0.367 gl · L^−1^ DOTA-bombesin derivative (BD) (signal intensity was reduced 10-fold in the graph), raw PAA nanogels (NG), and of the product—nanocarriers obtained by coupling PAA nanogels with DOTA-bombesin derivative, NGBD100H (for sample codes, see Table 2).

The results from the absorption spectra were supplemented with fluorescence measurements. First, the substrates used for coupling were measured. The maximum intensity on the fluorescence emission spectra of DOTA-bombesin derivative was at ca. 350 nm (see Figure 7), which corresponds to the tryptophan maximum at ca. 350 nm in water [96,97]. The spectrum of DOTA-bombesin derivative (concentration 0.367 gl · L^−1^) was reduced 10-fold for clarity (BD10×). The emission intensity of neat PAA nanogels (concentration 1.01 gl · L^−1^) was minor in comparison to other samples. The spectrum of nanogels derivatized with DOTA-bombesin NGBD100H (a representative sample) exhibited a relatively strong signal with a maximum at 350 nm, thus confirming successful coupling. These measurements confirm that the conjugation process was successful; however, quantitative determination of BN in the samples was not attempted, considering the lack of additivity of fluorescence.

The fluorescence decays for the abovementioned systems were biexponential, with almost the same lifetimes and amplitudes. The average fluorescence lifetime was in the range of 1.83–1.92 ns and was shorter than that measured for L-tryptophan (2.64 ns). The two lifetimes of the fluorescence of tryptophan are due to two sub-structures existing in its excited state and are intrinsic to tryptophan independently of the solvent. It seems that tryptophan moiety within nanogels is not significantly affected by the local microenvironment.

**Figure 8:**
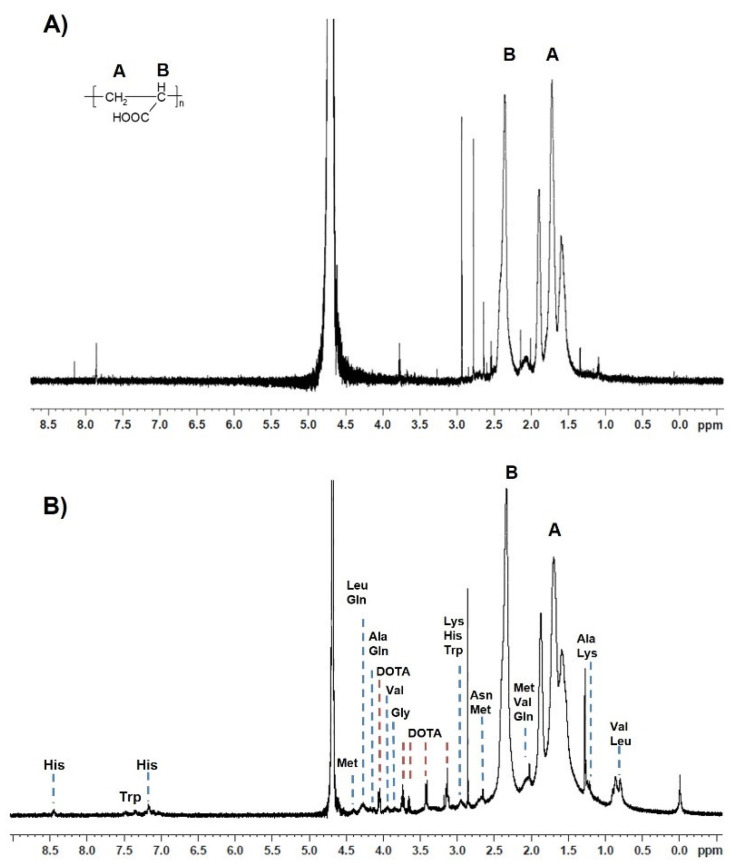
^1^H NMR spectra of (**A**) poly(acrylic acid) nanogels, (**B**) nanogels of PAA/bombesin—DOTA derivative in the molar ratio of 100/1, sample NGBD100E, recorded in D_2_O, 700 MHz. Single amino acids signals are presented with blue dashed lines and DOTA signals with brown dashed lines.

In Section 3.2, the ^1^H NMR spectrum of neat PAA nanogels was compared with the spectra of nanogels coupled with the model compound. Successful coupling was confirmed by the presence of signals from p-toluidine protons of the aromatic group visible above 7 ppm. Here the spectrum of raw nanogels (Figure 8A) was compared with nanogels coupled with bombesin—DOTA derivative in the molar ratio of 100/1, sample NGBD100E (Figure 8B). The highest intensity signals were from the CH_2_ (d, 1.70 ppm) and CH groups (t, 2.33 ppm) of the PAA nanogels. The signals from the aliphatic chain of oligopeptide (in the range 0.7–4.5 ppm) and DOTA bands (near 3–4 ppm) were partially overlayed by poly(acrylic acid); therefore, it was difficult to assign all the signals corresponding to the appropriate protons. In the literature, the ^1^H NMR spectra of bombesin [98,99] and DOTA [100] and the signal assignments are well known. Based on that, despite the low intensity of bombesin- and DOTA-derived signals on our spectrum, it was possible to identify many of them. The most characteristic bands were those derived from aromatic His-12 (2H, s, 8.45 ppm and 4H, s, 7.17 ppm) and Trp-8 (many signals from 2H, s, 5H, d, 6H, t, 7H, t, 8H, d, all in the range of 7.15–7.45 ppm). On the basis of the obtained spectrum, the following amino acid signals could be read: Met-14 (α-CH, t, 4.40 ppm), Leu-13 and Leu-4 (α-CH, t, 4.40 ppm), Gln-2 (α-CH, t, 4.36 ppm), Ala-9 (α-CH, quartet, 4.20 ppm), Gln-7 (α-CH, t, 4.10 ppm), Val-10 (α-CH, d, 4.00 ppm), Gly-5 and Gly-11 (α-CH, s, 3.85 ppm), His-12 (β-CH_2_, d, 3.10 ppm), Trp-8 (β-CH_2_, d, 3.08 ppm), Lys-1 and Lys-3 (ε-CH_2_, t, 2.98 ppm), Asn-6 (β-CH_2_, d, 2.69 ppm), Met-14 (γ-CH_2_, t, 2.60 ppm; ε-CH_3_, s, 2.08 ppm), Val-10 (β-CH, o, 2.05 ppm), Gln-2 and Gln-7 (β-CH_2_, quartet, 2.00 ppm), Ala-9 (β-CH_3_, d, 1.30 ppm), Lys -1 and Lys-3 (γ-CH_2_, quintet, 1.35 ppm), Val-10 (γ-CH_3_, d, 0.90 ppm), and Leu-4 and Leu-13 (δ-CH_3_, d, 0.80–0.90 ppm). Signals from the DOTA protons were visible between the amino acids in the region of 3–4 ppm: CH_2_ groups inside the ring (t, 3.73 ppm, t, 3.65 ppm, t, 3.12–3.18 ppm) and α-CH groups next to carboxylic groups (s, 4.06 ppm, s, 4.04, s, 3.43 ppm, s, 3.41 ppm).

### 3.4. Radiolabeling of DOTA-Bombesin Derivative

The radiolabeling study revealed that PAA nanogels coupled with derivative could be effectively labeled with lutetium-177. All of the NGBD derivatives were studied for labeling in the same conditions. The summarized results of the study are presented in Table 7.

**Figure 9:**
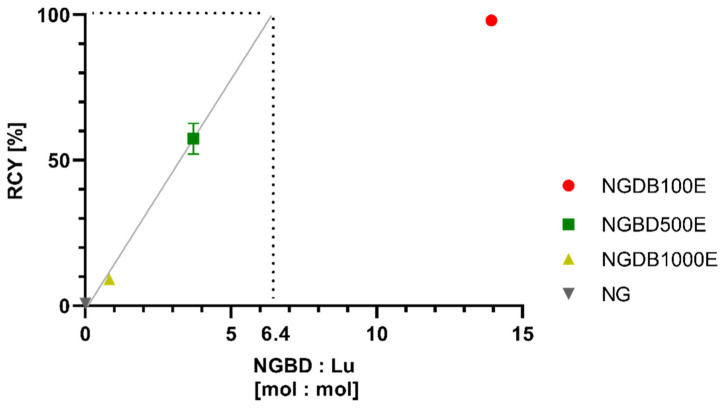
Lutetium-177 radiolabeling yield of NGBD particles (0.73 +/− 0.04 GBq mg^−1^ NGBD) in the function of the calculated BD-to-Lu ratio. The figure presents the estimation of the optimal NGBD-to-Lu ratio of 6.4, enabling radiolabeling with 100% yield.

The amount of lutetium-177 used for the NGBD labeling was constant for all labeling experiments calculated as 1:1, basing on the expected molar amount of bombesin conjugated to the NGBD1000E nanoparticles. In theory, assuming ideal conditions (no DOTA conjugating metals competing with lutetium and no steric hindrance), the labeling yield for NGBD1000E should be near 100%. In the experiment, a good >95% radiolabeling yield was achieved for NGBD100E, where the BD’s molar ratio to lutetium was almost 14. Basing on the experimental data, the optimal ratio of the chelating moiety to the lutetium (around 6.5) was calculated based on the linear regression equation. The curve is presented in Figure 9.

The lower-than-expected labeling yield may have been caused by some steric hindrances affecting accessibility for the radioisotope of the DOTA chelator on the bombesin chain. Another possible cause of the decreased labeling yield may have been a few labeled samples being contaminated with a trace amount of metals such as iron, copper, lead, or zinc.

The labeling of native nanogels (RCY = 0%) confirmed the specificity of the labeling with the attached BD. The reaction confirmed that weak interactions between carboxyl groups of nanogel, as well as the sponge-like structure of particles, do not lead to non-specific ^177^Lu uptake.

In the second part of the labeling experiments, the labeling of NGBD100E with increasing [^177^Lu]LuCl_3_ specific activity (SA) was studied. The results confirmed previous findings that the labeling yield decreases with the increase in activity used. When the molar excess of conjugated BD to lutetium was 2.5, the labeling yield decreased to 20%. In that case, high specific activity of 2.7 GBq mg^−1^ nanoparticles could only be achieved after purification of the labeling mixture. The maximum SA that could be achieved without purification was ca. 1 GBq mg^−1^ of NGBD100E. The summarized data are presented in Table 8.

## 4. Conclusions

Despite significant developments in the field of cancer management, there is a constant demand for better diagnostic and treatment options. New perspectives open along with the development of nanotechnology, as nanomaterials have the potential to improve the properties of the currently used drugs (i.e., their solubility and stability), prolong the time they reside in the bloodstream, and help to reduce the side effects of the therapy. Moreover, nanomaterials offer remarkable added value—sophisticated design, including diagnostic modalities, may render these formulations theranostic, i.e., combine diagnosis and treatment.

The presented work summarizes the efforts aimed at the application of radiation-derived poly(acrylic acid) nanogels as targeted nanocarriers of theranostic radioisotopes. The challenge of coupling nanogels with DOTA-bombesin derivative was successfully approached by the use of an innovative coupling strategy, designed according to the concept of “superactive esters,” based on DMT/NMM/TsO^−^. This strategy was also proven to be suitable for the complete in-house synthesis of our advanced targeting ligand. The obtained DOTA-bombesin nanocarriers showed excellent physicochemical properties and radiolabeling characteristics—high radiochemical yield was achieved upon complexation of lutetium-177 by the DOTA present in the nanocarrier. Overall, the presented research provides a proof-of-concept for the synthesis and functionalization of radiation-derived nanogel for theranostic applications and paves the way to further development for pre-clinical use.

## Figures and Tables

**Table 1:** Molar ratios between carboxylate groups of PAA nanogels (NG), DMT/NMM/TsO^−^ (ca), and p-toluidine molecules (T).

Sample	NG Carboxylate Groups	DMT/NMM/TsO^−^	p-Toluidine
NG/ca/T 1/1/1	1	1	1
NG/ca/T 2/1/2	2	1	2
NG/ca/T 10/1/10	10	1	10
NG/ca/T 10/10/1	10	10	1

**Table 2:** Nanocarriers obtained from coupling PAA nanogels and bombesin derivatives.

Name of the Sample	Molar Ratio		Source and Type of Bombesin Derivative
	Carboxylate Groups of PAA Nanogel	DOTA-Bombesin Derivative	
NGBD100H	100	1	in-house synthesized, with DOTA
NGB100H	100	1	in-house synthesized, without DOTA
NGBD100E	100	1	supplied by external company, with DOTA
NGBD500E	500	1	supplied by external company, with DOTA
NGBD1000E	1000	1	supplied by external company, with DOTA

Abbreviations: NGBD—nanogels conjugated with BD; NGB—nanogels conjugated with bombesin derivative; 100/500/1000—molar ratio of carboxylic groups to peptide; H—in-house synthesized peptide; E—externally supplied peptide.

**Table 3:** Molecular parameters: weight-average molecular weight (*M_w_*), radius of gyration (*R_g_*), hydrodynamic radius (*R_h_*), and density of polymer coil (*ρ_coil_*) of non-irradiated and irradiated PAA solutions. All these parameters were measured in an aqueous solution of 0.5 mol · dm^−3^ NaClO_4_, pH 10.

Sample	*M_w_* [Da = g · mol^−1^]	*R_g_* [nm]	*R_h_* [nm]	*ρ_coil_* [g · mol^−1^ nm^−3^]
Non-irradiated polymer	(8.87 ± 1.55) × 10^5^	115 ± 17	42 ± 3	0.14 ± 0.04
Nanogels irradiated at 5.3 kGy (NG_I)	(1.26 ± 0.09) × 10^6^	94 ± 5	45 ± 4	0.36 ± 0.07
Nanogels irradiated at 5.4 kGy	(1.13 ± 0.11) × 10^6^	86 ± 22	35 ± 1	0.43 ± 0.31
Nanogels irradiated at 5.4 kGy and re-filtered (NG_II)	(3.83 ± 0.34) × 10^5^	41 ± 2	30 ± 4	1.35 ± 0.28

**Table 4:** Z-average hydrodynamic radius (±standard deviations) for raw nanogels (NG_I) and nanogels coupled with p-toluidine. All samples were measured after dialysis. pH of the samples during the measurement was in the range of 3.9–4.3.

Sample	Z-Average *R_h_* (nm)
NG_I	186 ± 19
NG/ca/T 1/1/1	127 ± 18
NG/ca/T 2/1/2	109 ± 7
NG/ca/T 10/1/10	100 ± 9
NG/ca/T 10/10/1	105 ± 3

**Table 5:** The values of the Z-average hydrodynamic radius (± standard deviations) for raw nanogels and nanogels coupled with bombesin-DOTA derivative. All samples were measured after dialysis. “E” series products are based on NG_I batch, “H” series on the NG_II batch of nanogels.

Sample	Z-Average *R_h_* [nm]
NG_I	186 ± 19
NGBD100E	212 ± 36
NGBD500E	176 ± 19
NGBD1000E	217 ± 25
NG_II	113 ± 43
NGBD100H	102 ± 9
NGB100H	145 ± 1

**Table 6:** The efficiency of the coupling process estimated from the UV-Vis data. Different expected bombesin concentration values for samples with the same molar ratio result from differences in the volume of the solution (dialysis effect).

Sample	Expected Bombesin Concentration at 100% Efficiency (mg/mL)	Estimated Bombesin Concentration from UV-Vis Data (mg/mL)	Approximate Coupling Efficiency (%)
NGBD100E	0.281	0.252	90
NGBD500E	0.061	0.067	111
NGBD1000E	0.031	0.015	47
NGBD100H	0.313	0.203	65
NGB100H	0.202	0.105	52

**Table 7:** The radiochemical yield (%) of the PAA nanogel samples coupled with DOTA-bombesin derivatives and raw nanogels after the purification process.

	NGBD100E	NGBD500E	NGBD1000E	NG
NGBD:Lu [mol:mol]	13.9	3.7	0.8	0
[^177^Lu]Lu-sample	98.1 ± 0.7	57.4 ± 5.3	8.1 ± 0.6	0.7 ± 0.1
[^177^Lu]Lu-BD	0.0 ± 0.0	0.0 ± 0.0	0.0 ± 0.0	0.0 ± 0.0
[^177^Lu]Lu free	2.0 ± 0.6	42.6 ± 5.3	91.9 ± 0.6	99.3 ± 0.1

**Table 8:** Radiochemical yield (%) as a function of SA for sample PAA nanogels coupled with DOTA-bombesin derivative in a molar ratio of 100/1 (sample NGBD100E).

	Activity for Labeling (GBq mg^−1^)				
	19.64	9.31	0.78	0.68	0.18
BD:Lu [mol:mol]	1.3	2.5	21.7	56.5	141
[^177^Lu]Lu-NGBD100E [%]	13.8 ± 1.2	20.2 ± 1.7	99.0 ± 1.1	98.1 ± 0.7	99.5 ± 0.7
SA [GBq mg^−1^]	2.71	1.88	0.77	0.67	0.18

## Data Availability

The data presented in this study are available on request from the corresponding author.
